# Degradation of CaMKII is stimulated by its active conformation

**DOI:** 10.1016/j.jbc.2025.110601

**Published:** 2025-08-14

**Authors:** Christina Quasney, Leslie C. Griffith

**Affiliations:** Department of Biology, Program in Biochemistry and Volen National Center for Complex Systems, Brandeis University, Waltham, Massachusetts, USA

**Keywords:** Ca2+/calmodulin-dependent protein kinase II (CaMKII), *Drosophila*, ubiquitin, protein degradation, autophosphorylation, HEK293T cells

## Abstract

Maintaining synaptic protein levels that support both functional stability and plasticity is crucial for the proper function of the nervous system. Calcium/calmodulin-dependent protein kinase II (CaMKII) plays a pivotal role in synaptic signaling, with its synaptic levels regulated by both activity-dependent translocation from the cytosol and local translation. However, the mechanisms governing the removal of CaMKII from synapses and its degradation remain poorly understood. In this study, we develop a HEK293T cell culture system to investigate what properties of CaMKII regulate its degradation. By isolating distinct conformational states of both *Drosophila* and rat CaMKII, we demonstrate that locking CaMKII in its active form *via* a phosphomimetic mutation at the autonomous activation site significantly reduces the half-life for both homologs. We identify ubiquitination as the primary degradation pathway for persistently active CaMKII mutants. Moreover, our findings suggest that the decreased half-life is primarily due to the exposure of the catalytic and regulatory domains, rather than changes in the oligomerization state of the holoenzyme or regulation of degradation pathways by the kinase's enzymatic activity. These results provide a template for understanding CaMKII degradation in neurons.

Calcium/calmodulin-dependent protein kinase II (CaMKII) is one of the most abundant proteins present in neurons. In the rodent hippocampus, it has been estimated to account for 2% of total protein and is highly enriched at synapses across phyla (1, 2). CaMKII has critical roles in multiple neuronal processes and is a key regulator of synaptic plasticity and memory formation in both mammals and *Drosophila* (3, 4). Despite its importance, how its steady-state levels are regulated is not completely understood.

CaMKll exists primarily as a holoenzyme consisting of 12 subunits arranged as a hexamer of vertical dimers. Each subunit has three functional domains: catalytic, regulatory, and association. The N-terminal catalytic domain houses residues critical for the coordination of ATP, binding of substrate, and transfer of gamma phosphate to substrate. The regulatory domain contains partially overlapping auto-inhibitory and calmodulin-binding regions and lies between the catalytic and association domains. The C-terminal association domain is responsible for its multimeric holoenzyme structure. These three primary domains are highly conserved across species, with the most variance arising from a flexible linker that is positioned between the regulatory and association domains (5, 6).

The regulation of the activity and conformational states of the CaMKII holoenzyme is complex. In a low-Ca^2+^ environment, the regulatory domain of each subunit is docked at the substrate-binding site of its catalytic domain. When intracellular calcium levels rise, binding of Ca^2+^/calmodulin displaces the autoinhibitory segment of the regulatory domain from the catalytic site, exposing the substrate-binding pocket as well as T286 (T287 in *Drosophila*), a key, highly conserved residue located at the hinge between the two domains. Once exposed, T286 can undergo *trans* autophosphorylation by a neighboring Ca^2+^/calmodulin-bound subunit. Autophosphorylation at T286 prevents reassociation of the regulatory and catalytic domains, causing the holoenzyme to lose its compact autoinhibited structure and adopt a more extended conformation (7, 8). In this state, the enzyme is autonomously active; the catalytic domain binds substrates without requiring Ca^2+^/calmodulin. The regulatory domain is now exposed for interactions with other proteins or subsequent phosphorylation at the T305/T306 (T306/T307 in *Drosophila*) sites within the CaM-binding domain. Phosphorylation of these sites blocks Ca^2+^/calmodulin re-binding and has been shown to facilitate subunit exchange in the otherwise stable holoenzyme (9).

The cell biology of CaMKII is also complex and dynamic. Activation of the enzyme in neurons by calcium influx is associated with the translocation of cytoplasmic protein to synaptic complexes (10). In both rodent and *Drosophila* neurons, activity can also increase overall levels of CaMKII by stimulating local translation of synaptically localized mRNA (11, 12). This increase in the amount of synaptic CaMKII is an early required step for several different types of plasticity (1, 13, 14), which in mammals are associated with morphological changes such as the expansion of synaptic area and the formation of new synaptic connections (15, 16).

While activity-dependent addition of CaMKII to synapses is clearly a key part of synaptic plasticity, there are biological limitations. Unopposed activity-dependent addition of proteins to synaptic complexes could, in theory, quickly saturate binding sites and block further plastic change. Neurons are physical entities, and expanding synaptic complexes compete for limited real estate; crowding could have negative effects on signaling specificity and network function. At a more global level, optimum operation of the nervous system is thought to require homeostatic regulation of processes modulating synaptic strength to maintain stable network changes while still allowing ongoing plasticity (17). These mechanistic and spatial considerations suggest that there must be complementary processes that remove and/or degrade CaMKII from synapses to prevent saturation of biochemical interactions and overgrowth of synapses.

The logical counter to new protein synthesis at synapses is regulated protein degradation (18, 19). Consistent with this idea, many studies have shown that the ubiquitin-proteasome system, one of the most important cellular protein degradation pathways, is critical for the formation of long-term memory (20), and induction and maintenance of LTP and synaptic remodeling (21, 22, 23). Consistent with a role in plasticity, neuronal proteasomes are regulated by activity in terms of both function and localization (24, 25). While regulatory ubiquitination of CaMKII has been demonstrated (26, 27), what controls the degradation of CaMKII is still unknown, though proteomic studies of neuronal ubiquitin ligases show that they can associate with CaMKII (28).

In this study, we develop a cell culture pulse-chase assay in HEK293T cells to address how the half-life of CaMKII is regulated. We exploit half-life and sequence differences between rat CaMKIIα and the *Drosophila* CaMKII to map the structural determinants of stability and show that conformational changes associated with activation are associated with enhanced ubiquitination and degradation of the protein.

## Results

**Figure 1:**
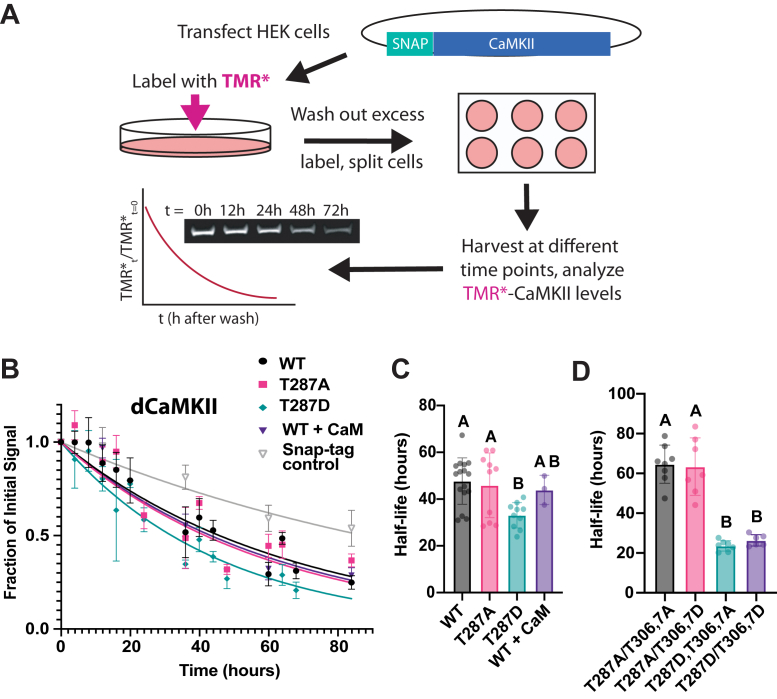
**Half-life of *Drosophila* CaMKII is regulated by the autonomy site T287.***A*, schematic of HEK293 cell pulse-chase assay. *B*, determination of the half-life of WT and T287 mutant *Drosophila* CaMKII. Fraction of labeled dCaMKII constructs (WT, T287A, T287D, WT + CaM, and the SNAP-tag-only control) plotted over time. Data represents the mean normalized signal (T/T_0_) at each time point, with error bars indicating the standard deviation from the mean (SD) across measurements. N = 16 for WT; 10 for T287A; 10 for T287D, three for WT + CaM; five for SNAP-tag alone. Decay curves were fitted using the averaged decay rate for each construct. *C*, the active conformation (T287D) of *Drosophila* CaMKII decreases half-life. Half-life was calculated from fitted decay rates. Data are presented as mean ± SD. Statistical analysis was performed using one-way ANOVA with Tukey’s multiple comparisons test: WT *versus* T287A, *p* = 0.9703; WT *versus* T287D, *p* = 0.0051; T287A *versus* T287D, *p* = 0.0352; WT *versus* WT + CaM, *p* = 0.9328; T287A *versus* WT + CaM, *p* = 0.9907; T287D *versus* WT + CaM, *p* = 0.3713. N = 16 for WT; 10 for T287A; 10 for T287D, three for WT + CaM. *D*, Autophosphorylation within the calmodulin-binding domain does not contribute to activation-dependent degradation. Half-life was calculated from fitted decay rates. Data are presented as mean ± SD. One-way Anova with Tukey’s multiple comparisons of the activation state mutants. T287A,T306,7A *versus* T287A/T306,7D, *p* = 0.9935; T287A,T306,7A *versus* T287D,T306,7A, *p* < 0.0001; T287A,T306,7A *versus* T287D/T306,7D, *p* < 0.0001; *versus* T287A,T306,7A *versus* T287D/T306,7A, *p* < 0.0001; T287A/T306,7D *versus* T287D/T306,7A, *p* < 0.0001; T287A/T306,7D *versus* T287D/T306,7D, *p* < 0.0001; T287D,T306,7A *versus* T287D/T306,7D, *p* = 0.9452. N = 8 for T287A/T306,7A; seven for T287A/T306,7D; seven for T287D/T306,7A, six for T287D/T306,7D. Statistical differences are indicated by groups in panels C and D.

To measure the half-life of CaMKII, we transfected SNAP-tag N-terminal fusions of the *Drosophila* (NP_524635, PC isoform, 490 aa; hereafter dCaMKII) or rat CaMKIIα (NM_012920.1, 30 aa linker isoform, 478 aa; hereafter rCaMKII) into HEK293T cells. These two CaMKII proteins are both abundant isoforms in their native tissues with similarly sized, minimal variable linkers (29, 30). The proteins are overall 77% identical and 87% similar, highlighting the strong conservation of this enzyme. CaMKII was labeled with a pulse of TMR-Star, a cell-permeable fluorescent SNAP-ligand. Excess dye was washed away to prevent labeling of protein synthesized after the pulse. Cell extracts were collected at different time points and analyzed by SDS-PAGE (31, 32). The fraction of remaining TMR-Star signal at the molecular weight of the SNAP::CaMKII monomer over time was plotted and fitted to a one-phase exponential decay, and the corresponding half-lives were calculated from the extracted decay rate (Fig. 1*A*). This type of assay has been shown to be an effective and powerful way to study the half-life of a protein (33).

The first question we wanted to ask was whether CaMKII degradation was affected by autophosphorylation. To investigate this we made point mutations at T287 to generate phosphonull (T287A) and phosphomimetic (T287D) versions of the *Drosophila* kinase with the SNAP-tag fusion. Since we did not know the stoichiometry or dynamics of phosphorylation of this site in HEK293T cells, the fact that the point mutant kinases were by definition 100% "phospho" or "dephospho" for each of the particular sites we wanted to study allowed cleaner interpretation of results. We measured the amount of TMR-Star-labelled protein to calculate half-lives for WT, T287A, and T287D dCaMKII. As a control, we examined the half-life of the SNAP-Tag protein itself. Figure 1*B* shows raw data plotted as a fraction of initial protein remaining over time. The half-life of the SNAP-tag itself was significantly longer than that of any of the dCaMKII proteins, indicating that the tag does not stimulate the degradation of the fusion proteins. Half-life of the dCaMKII proteins is plotted in Figure 1*C*. The autonomously active point mutant T287D had a significantly shorter half-life compared to either the WT or the T287A protein.

While this result suggested that autophosphorylation might have an impact on half-life, it was notable that the WT and T287A dCaMKII were not significantly different. One reason for this could be that the endogenous pT287 stoichiometry might be low. Another possibility could be that since HEK293T cells are known to have low levels of endogenous calmodulin (CaM) (34), the overexpressed WT dCaMKII might be outstripping the amount of available CaM and not getting autophosphorylated. To determine if CaM could enhance WT dCaMKII degradation, we carried out a set of experiments in which we co-transfected with equal amounts of CaM-encoding plasmid. Comparison WT half-life with WT + CaM (Fig. 1, *B* and *C*) shows no difference. We conclude that the level of endogenous CaM does not affect measured half-life in this assay system and that the resting level of pT287 is likely to be low in these cells for other reasons.

Loss of autoinhibition after phosphorylation at T287 has several consequences for CaMKII. One important one is that the kinase can now autophosphorylate additional sites in its regulatory domain when Ca^2+^/calmodulin dissociates. To determine if these sites were important for regulation of degradation we made both phosphomimic and phosphonull substitutions at these threonines and asked if they affected the half-life of either T287A or T287D dCaMKII. Figure 1*D* shows calculated half-life for the triple mutants. Mutation of T306/7 had no effect on the stability of either T287A or T287D kinases. This suggests that the phosphorylation status of T287 is a major driver of dCaMKII degradation.

**Figure 2:**
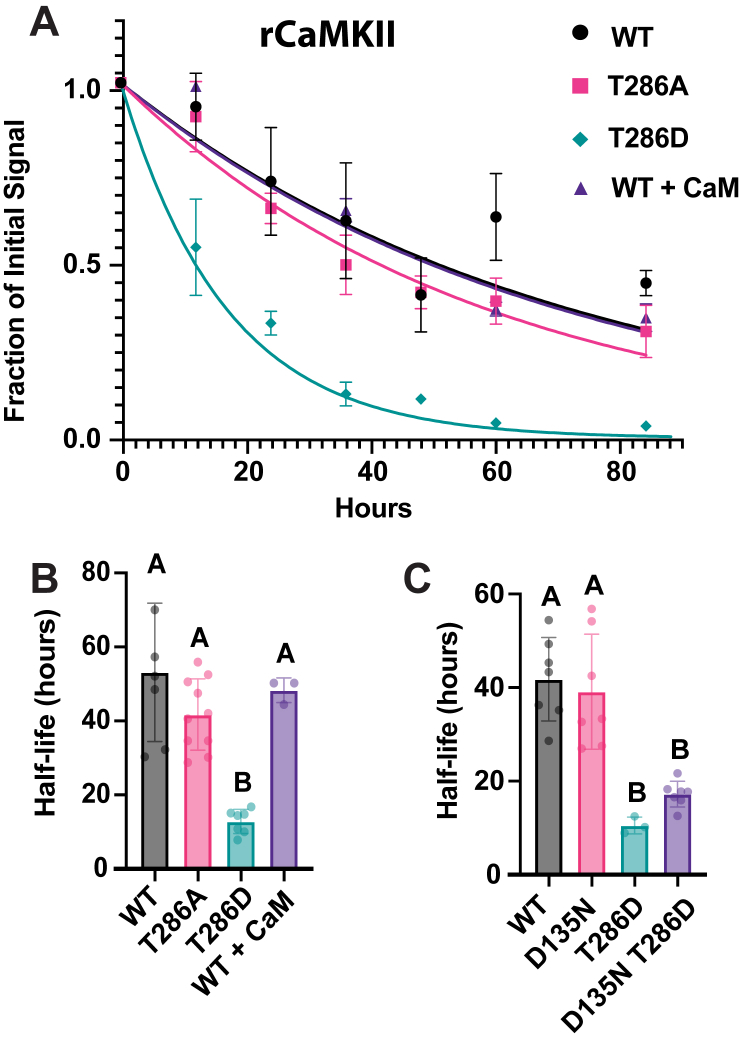
**Half-life of mammalian CaMKII is regulated by the autonomy site T286.***A*, determination of the half-life of WT and T286 mutant rat CaMKII. Fraction of labeled rCaMKII (WT, T286A and T286D) plotted over time. Data points represent the mean normalized signal (T/T_0_) at each time point, with error bars reporting SD from the mean. N = 7 for WT; 10 for T286A; seven for T286D, three for WT + CaM. Decay curves were fitted using the averaged decay rate for each construct. *B*, The active conformation of rCaMKII has a decreased half-life. Half-life comparisons for all constructs were calculated from the fitted rate of decay. Data represent mean ± SD. One-way ANOVA with Tukey’s multiple comparisons test, WT *versus* T286A, *p* = 0.2085; WT *versus* T286D, *p* < 0.0001; WT *versus* WT + CaM, *p* = 0.9269; T286A *versus* T286D, *p* = 0.0002; T286A *versus* WT + CaM, *p* = 0.8188; T286D *versus* WT + CaM, *p* = 0.0009. N = 7 for WT; 10 for T286A; seven for T286D, three for WT + CaM. Data for SNAP-tag alone demonstrating that it is more stable than rCaMKII is shown in Figure 1*B*. *C*, kinase activity is not required for the T286D mutation to accelerate degradation. Half-life comparisons for all constructs were calculated from the fitted rate of decay. Data represent mean ± SD. One-way ANOVA with Tukey’s multiple comparisons test, WT *versus* D135N, *p* = 0.9359; WT *versus* T286D, *p* = 0.0002; WT *versus* D135N T286D, *p* = 0.0001; D135N *versus* T286D, *p* = 0.0005; D135N T286D *versus* T286D, *p* = 0.6664; D135N *versus* D135N T286D, *p* = 0.0005. N = 7 for WT; seven for D135N; three for T286D, seven for D135N T286D. Statistical differences are indicated by groups in panels B and C.

To ask if this finding was a conserved feature of CaMKII biology, we examined the stability of rat αCaMKII in our pulse-chase system. Figure 2*A* shows raw data for WT, WT + CaM, T286A, and T286D rCaMKII. Similar to the *Drosophila* protein, mutation of T286 to mimic phosphorylation and render the enzyme Ca^2+^-independent accelerated degradation. WT and T287A were indistinguishable, and WT degradation was not affected by the addition of more CaM. Degradation of the SNAP-tag alone (shown in Fig. 1*B*) was slower than any of the rCaMKII proteins.

As noted above, mutation of the autonomy site of CaMKII to a phosphomimic residue can allow secondary autophosphorylations within the CaMKII subunit. It can also increase the phosphorylation of cellular substrate proteins since the enzyme no longer requires Ca^2+^/calmodulin. Since CaMKII has been suggested to positively regulate proteasome activity (25, 35), this raised the possibility that the decreased stability of the autonomous enzymes was due to upregulation of some degradation pathway. To determine if either of these scenarios was important for the degradation of rCaMKII, we asked if kinase activity was required for the accelerated degradation of T286D rCaMKII by introducing a mutation in the catalytic domain (D135N) that blocks enzyme activity (36). Figure 2*C* shows data for D135N rCaMKII and the double mutant D135N, T286D. Dead kinase with the T286D mutation was significantly less stable than dead kinase without the mutation of the autonomy site. Statistical comparison of the half-lives of WT to D135N and T286D to D135N, T286D showed that the kinase-dead proteins were indistinguishable from the parent proteins. We conclude that the T286D mutation is driving degradation of rCaMKII, not additional autophosphorylation or phosphorylation of other cellular proteins.

**Figure 3:**
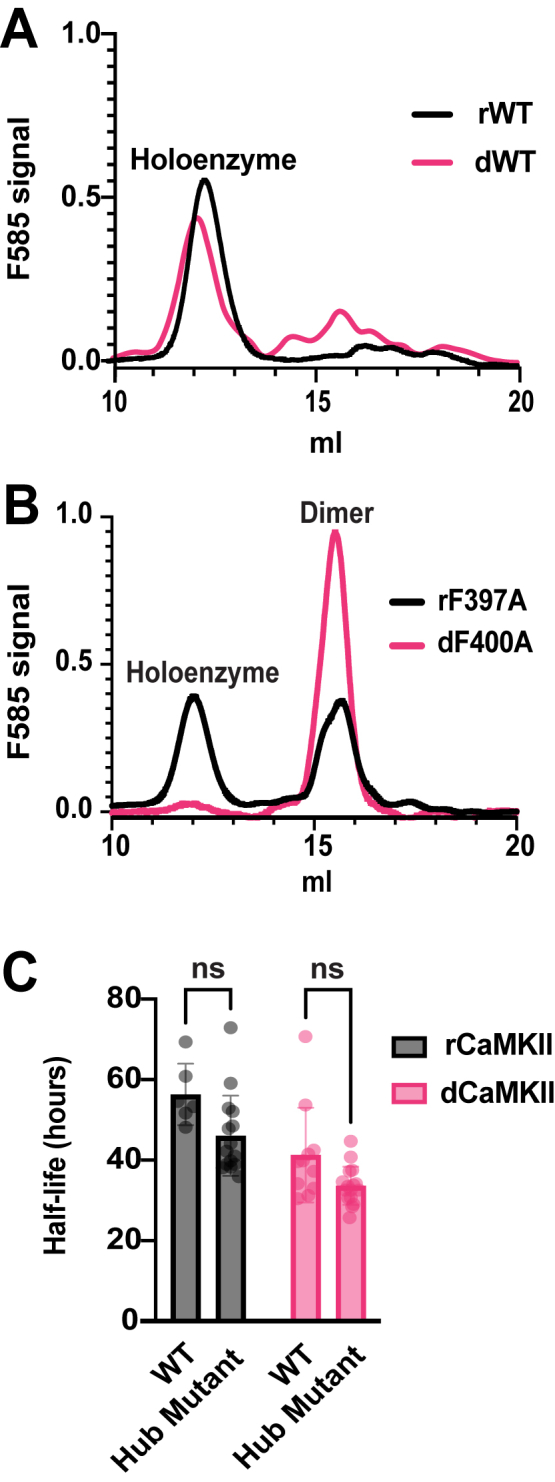
**Dimer release does not significantly contribute the decreased half-life of activated CaMKII.***A*, chromatogram of WT rCaMKII (*black*) and WT dCaMKII (*pink*). The peaks are normalized to total area under the curve for comparison. *B*, chromatogram of rCaMKll F397A mutant (*black*) and corresponding dCaMKll F400A (*pink*) mutant. The peaks are normalized to total area under the curve for comparison. *C*, Analysis of the effect of destabilizing mutations in the hub on the half-life of rCaMKII (*black*) and dCaMKII (*pink*). Grouped comparison of WT *versus* hub mutants for both rCaMKII and dCaMKII. Statistical significance was assessed using an unpaired *t* test with Bonferroni correction for multiple comparisons. The comparison of WT rCaMKII *versus* F397A rCaMKII yielded *p* = 0.0715, and WT dCaMKII *versus* F400A dCaMKII yielded *p* = 0.0541. N = 6 for rCaMKII WT; N = 15 for rCaMKII hub mutant; N = 11 for dCaMKII WT; N = 16 for dCaMKII hub mutant.

Phosphorylation at the T286 autonomy site has also been shown to stimulate subunit exchange in the CaMKII holoenzyme (9). This process is believed to occur when the autonomous enzyme phosphorylates residues in the CaM-binding domain (T305,6). These phosphorylated residues destabilize the hub to allow release of vertical dimers (37). This raised the possibility that the quickly degraded form of the kinase is dimers that have been released from the activated holoenzyme rather than the holoenzyme itself. To test this idea, we examined the half-life of CaMKII containing destabilizing hub mutations: F397A rCaMKII (37) and F400A dCaMKII. We verified the release of dimer species using analytical gel filtration. Compared to WT CaMKIIs, which size predominantly as dodecamers (Fig. 3*A*, Table 1), the rCaMKII hub mutant in crude cell extracts sized as half dodecamer and half dimer, while the fly hub mutant was essentially all dimer (Fig. 3*B*, Table 1). Comparison of the half-lives of these hub mutants to their cognate WT enzymes demonstrated that although they appeared to be slightly shorter, they were not statistically significantly different from WT. Combined with our findings that 1) phosphomimic mutations within the calmodulin-binding domain do not affect stability, and 2) the amount of dimer is much greater for the dCaMKII hub mutant but difference in half-life between WT and hub mutant is the same for both species (Fig. 3*C*), this strongly argues that activation-dependent release of dimers is not an obligate step in CaMKII degradation or the major determinant of CaMKII half-life.

**Table 1:** Gel filtration estimation of SNAP::CaMKII oligomer size

State	Construct	Predicted kDa	K_av_	Calculated kDa
Monomer	rCaMKII WT	74		
Dimer	rCaMKII WT	148		
Holoenzyme	rCaMKII WT	888–1036	0.27	748
Monomer	rCaMKII F397A	74		
Dimer (left shoulder)	rCaMKII F397A	148	0.45	140
Dimer (right shoulder)	rCaMKII F397A	148	0.47	111
Holoenzyme	rCaMKII F397A	888–1036	0.25	890
Monomer	dCaMKII WT	75		
Dimer	dCaMKII WT	150		
Holoenzyme	dCaMKII WT	905–1055	0.24	965
Monomer	dCaMKII F400A	75		
Dimer	dCaMKII F400A	150	0.46	124
Holoenzyme	dCaMKII F400A	905–1055		

Summary of the oligomerization states and molecular weights (MW) of each SNAP::CaMKII construct. Column 1 presents the estimated oligomerization state, Column 2 lists the corresponding SNAP::CaMKII constructs, and Column 3 provides the predicted MW, calculated using the Expasy PI/Mw calculator tool (51). The calculated K_av_ for each peak, representing its most likely associated oligomerization state, is shown in Column 4. Column 5 displays the MW for each peak, determined using an equation derived from MW standards calibration.

Having ruled out secondary phosphorylations and release of dimers as important drivers of degradation of CaMKII, we suspected that the conformational changes induced by activation of the enzyme might be important for targeting the active kinase for disposal. Activation results in a large conformational change in CaMKII (8) and loss of contacts between the catalytic and regulatory domains that have been shown analytically (38), crystallographically (39), and in intact cells with FRET (7). In addition to facilitating autophosphorylation, ATP and substrate binding, this conformational change may allow the kinase to interact with distinct sets of binding partners specific for activation state (40, 41).

**Figure 4:**
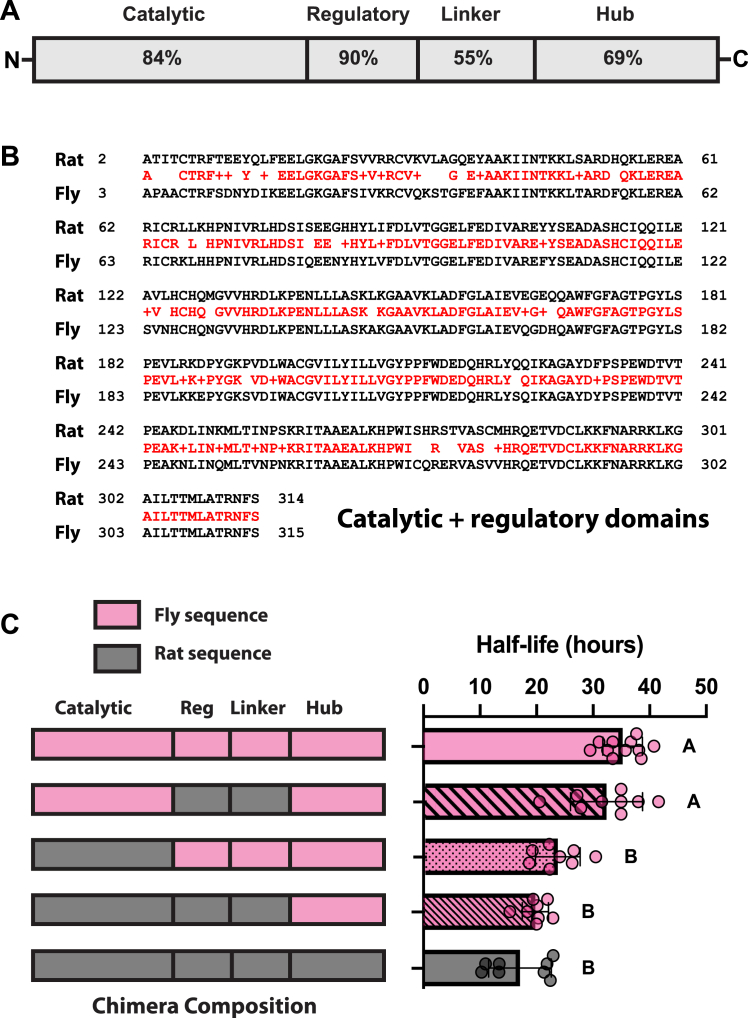
**Degradation is driven by exposure of the catalytic/regulatory domain interface.***A*, conservation across domains between rat and *Drosophila* CaMKII, derived from Smith-Waterman sequence alignment, expressed as percent sequence identity. *B*, alignment of rCaMKII (*Rat*) and dCaMKII (Fly) catalytic and regulatory domains. Alignment was carried out using the NCBI Blast tool. *C*, Schematic representation of full-length and chimera proteins (*left*). All proteins are autonomous phosphomimetic mutants (T286/7D). The fly sequence is represented in *pink* and the rat sequence in *black*. Full-length fly protein (T287D mutant) is shown at the top, with full-length rat protein (T286D mutant) at the bottom. The composition of each chimeric protein is depicted by the color of its domains. Half-lives are shown on the *right*. Comparisons were performed using one-way ANOVA with Tukey’s multiple comparisons test. The rat regulatory and variable linker domains alone (r(Reg + Linker)) are insufficient to reduce the half-life of dCaMKII autonomous phosphomimetic mutants, whereas the catalytic domain alone (r(Cat)) significantly decreases the half-life. The addition of the regulatory and variable linker domains (r(Cat + Reg + Linker)) further decreases the half-life, similar to that of the full-length rat autonomous phosphomimetic. Statistical results: Full length fly *versus* rat, *p* < 0.0001; fly *versus* r(Reg + Linker), *p* = 0.5434; fly *versus* r(Cat), *p* < 0.0001; fly *versus* r(Cat + Reg + Linker), *p* < 0.0001; rat *versus* r(Reg + Linker), *p* < 0.0001; rat *versus* r(Cat), *p* = 0.0584; rat *versus* r(Cat + Reg + Linker), *p* = 0.7576; r(Cat) *versus* r(Reg + Linker), *p* < 0.0001; r(Cat) *versus* r(Cat + Reg + Linker), *p* = 0.5038; r(Reg + Linker) *versus* r(Cat + Reg + Linker), *p* < 0.0001. N = 11 for dCaMKII T287D; N = 9 for first chimera; N = 8 for second chimera; N = 8 for third chimera; N = 8 for rCaMKII T286D. Statistical differences are indicated by groups in *panel* C.

To probe the idea that this conformational change might help drive degradation, we took advantage of the finding that the half-life of T286D rCaMKII was significantly shorter than that of T287D dCaMKII in HEK293T cells. We reasoned that if we created chimeric proteins between these two kinases, we could identify the part of the rCaMKII protein responsible for its activation-dependent instability. Figure 4*A* shows the domain structure of CaMKII with the % identity between rCaMKII and dCaMKII for each domain. Both the catalytic and regulatory domains show very high conservation, while the linker and hub domains are more dissimilar. Figure 4*B* shows an alignment of the rat and *Drosophila* catalytic and regulatory domains. Half-lives for chimeric proteins are shown in Figure 4*C*, with cartoon representations of the chimeric proteins on the left, and half-life data on the right. Importantly, in all the chimeras, the autonomy site had a phosphomimic mutation to accelerate degradation. We started by substituting the rCaMKII regulatory and variable linker regions into the *Drosophila* protein. This did not significantly reduce the half-life of the protein, ruling out the highly variable linker as the major cause of the difference in degradation. In contrast, substitution of the highly conserved rCaMKII catalytic domain into dCaMKII resulted in a significant decrease in half-life that was further reduced in the chimeric protein with both the rCaMKII catalytic and regulatory domains, though not to the level of the intact activated rCaMKII. These data suggest that exposure of the interface between the rCaMKII catalytic and regulatory domains is likely the major determinant of its activation-dependent degradation.

**Figure 5:**
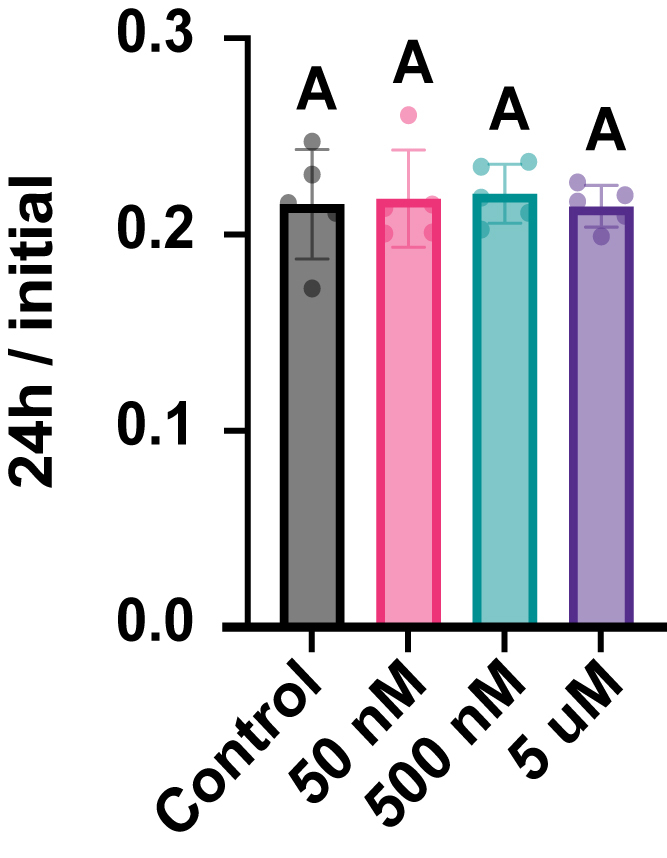
**Calpain cleavage does not regulate CaMKII degradation.** Comparison of the fraction of remaining labeled rat T286D CaMKII following 24 h treatment with the calpain protease inhibitor, Calpeptin, at concentrations of 50 nM, 500 nM, and 5 μM. No statistically significant differences were observed between Calpeptin-treated conditions and the control. Statistical analysis was performed using one-way ANOVA with Dunnett’s multiple comparisons test. Results: Control *versus* 50 nM, *p* = 0.9931; Control *versus* 500 nM, *p* = 0.9952; Control *versus* 5 μM, *p* = 0.9996. N = 5 for all conditions.

We next sought to determine the cellular pathway used for CaMKII degradation. Activation of CaMKII in neurons occurs in the context of elevated calcium, and in ischemic brain tissue, activation of calpain by calcium can cleave activated αCaMKII (42). To determine if the loss of CaMKII in HEK293T cells was calpain-dependent, we looked at the ability of Calpeptin, a potent and cell-permeable calpain inhibitor (ca. 40 nM; (43)), to block degradation of T286D rCaMKII. Transfected cells were labelled with TMR-Star and treated with various doses of calpeptin. The amount of T286D rCaMKII remaining at 24 h was assessed (Fig. 5). Calpeptin did not reduce CaMKII loss compared to the vehicle control, even at 5 μM. We conclude that calpain is not responsible for the activation-dependent loss of CaMKII in these cells.

**Figure 6:**
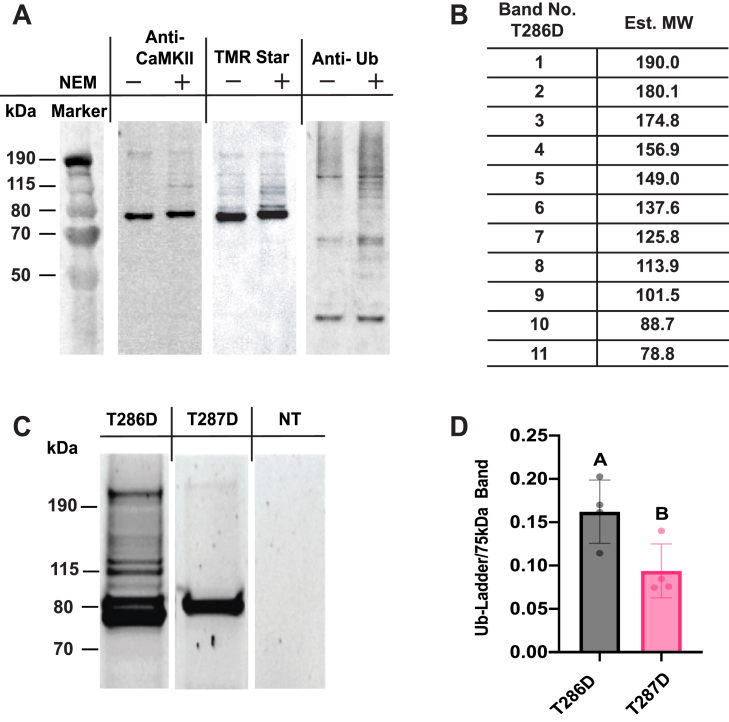
**Differential Ubiquitination of autonomous rat and *Drosophila* CaMKII in HEK293T cells.***A*, immunoblot analysis of partially purified T286D rCaMKII fractions from HEK293T cells following 48 h of expression and subsequent labeling with TMR-Star. The lysates on the *right* side of each *panel* are treated with 10 mM NEM, and the *left* side represents the untreated control. All three *panels* represent the same two gel lanes, blotted and probed for different things. *Left panel* demonstrates equal levels of CaMKII in both conditions, as indicated by the major ∼75 kDa band of the anti-CaMKII blot. *Center panel* shows the TMR-Star signal, with a more intense high MW band ladder in the +NEM fraction. *Right panel* shows an increase in anti-ubiquitin signal + NEM, as compared to the control -NEM condition. *B*, table summarizing MW shifts in the high MW ladder from a crude lysate of TMR-Star-labelled T286D rCaMKII. Average shift is 11.1 kDa. *C*, representative gel image showing lysates from HEK293T cells expressing either T286D rCaMKII (*left*) or T287D dCaMKII (*center*). The *right*-most *panel* is a non-transfected control lysate (NT). All lysates were labeled with TMR-Star. The mammalian CaMKII (rCaMKII) exhibits a more intense high MW ladder compared to *Drosophila* CaMKII (dCaMKII), suggesting a higher degree of ubiquitination. *D*, quantification of the TMR-Star signal from high MW bands of T286D rCaMKII and T287D dCaMKII, normalized to the main 75 kDa band. N = 4 for both groups.

A second candidate degradation pathway is the ubiquitin/proteasome system. In cultured cells, 80 to 90% of protein degradation occurs *via* this mechanism (44), and CaMKII is ubiquitinated in neurons (26, 27). This pathway is tightly regulated and relies on E3 ubiquitin ligases to recognize potential substrates, with subsequent poly-ubiquitination of lysine residues and targeting to the proteasome. To determine if CaMKII was a substrate for the proteasome, we asked if it was ubiquitinated. Since the steady-state levels of ubiquitinated species can be low, and the activity of deubiquinating enzymes is high, we partially purified CaMKII from our HEK293T cell lysates. Transfected T286D rCaMKII was labelled with a biotin-SNAP ligand spiked with a small amount of TMR-Star. Biotin-labeled holoenzymes were harvested on a streptavidin column, and TMR-Star-labelled subunits in the holoenzyme were visualized in this partially purified sample by SDS-PAGE. Figure 6*A* shows T286D rCaMKII extracts made ± 10 mM N-ethyl maleimide (NEM), an inhibitor of deubiquitinases (45). The TMR-Star label aligns with the anti-CaMKII-stained band at 75 kDa, the expected weight of the SNAP-tag T286D rCaMKII fusion protein. There is also a faint ladder of higher molecular weight bands above, and their intensity is increased by inclusion of NEM in the cell lysate, consistent with these bands representing poly-ubiquitinated rCaMKII species. To test this, we immunoblotted with an anti-ubiquitin antibody (Fig. 6*A*, right) to confirm an increase in ubiquitination for the +NEM condition. Given the distinct high-MW ladder above the main 75 kDa band in the T286D rCaMKll extract, we assessed the increments of molecular weight in a TMR-Star labelled lane of rCaMKII lysate. In this sample, the only TMR-Star-labelled species is rCaMKII, meaning that the ladder represents modified CaMKII. We found increments ranging from 5.6 to 17.9 kDa and averaging 11.1 kDa in the ladder, consistent with the incremental MW change that ubiquitination would impart (Fig. 6*B*).

We also compared activated rCaMKII to dCaMKII to determine if there was a difference in ubiquitination. Figure 6*C* shows partially purified protein visualized with TMR-Star. T286D rCaMKII again shows a clear ladder above the unmodified species. In contrast, while a similar amount of unmodified protein was pulled down, the ubiquitin ladder of T287D dCaMKII is very faint, though distinguishable from the non-transfected extract shown in the right-most panel. Quantification of the ratio of signal in the ladder normalized to the unmodified band from multiple independent experiments shows that dCaMKII has significantly lower amounts of higher MW bands (Fig. 6*D*). This suggests that the HEK293T cell ubiquitin-proteasome system recognizes activated rCaMKII better than the *Drosophila* enzyme, potentially explaining the difference in half-life between the two CaMKII proteins in this cell type.

## Discussion

In this study, we examine the mechanisms underlying CaMKII degradation using a cell culture pulse-chase paradigm. We exploit differences in half-life between rat and *Drosophila* CaMKII to determine that activation of the kinase, by exposing the interface normally hidden by interactions between the catalytic and regulatory domains, is a driver of CaMKII degradation. Further, we implicate the ubiquitin/proteasome system as a potential mediator of activation-specific degradation. Our finding that the activated conformation of CaMKII is more effectively eliminated suggests that degradation, like synaptic CaMKII synthesis (1, 11), is regulated by neuronal activity.

The use of a cell culture system with SNAP-tagged proteins provided several advantages. One major one was that we were able to label CaMKII with a very short pulse (30 min of label after 40 h of expression) and assay over a long (80 h) time course. For a long-lived protein like CaMKII, this is critical. A previous study in which 12 h metabolic labelling was used and cultured neurons harvested at 48 h did not detect changes in CaMKII half-life after bicuculline treatment (23), perhaps due to the relative lengths of the pulse and chase phases. The short time window of labelling and the pulse-chase design also make this type of assay robust to differences in transfection efficiency between constructs, since we are looking at the rate of decay and not protein levels, so there is no normalization required to make comparisons between constructs. The fact that we were able to use autonomously active point mutants that are 100% in the phospho-T286/7 conformation, as opposed to relying on long-term drug treatment to shift the balance of CaMKII autophosphorylation, likely also made it easier for us to see differences in half-life.

The cell culture approach for a protein like CaMKII also has some limitations. Firstly, the long half-life of CaMKII made using drugs with any level of toxicity (*e.g.* ones which disrupt ubiquitin/proteasome function or broadly block phosphatases) impossible because long time points became very unreliable in terms of cell recovery. The amount of cell death after 12 to 24 h prevent us from extending drug treatment to times that would have allowed us to see significant changes in half-life. Because we could not use standard proteasome inhibitors, this weakens the link to the ubiquitin system as the effector of degradation. While we show that CaMKII is ubiquitinated, ubiquitination is not always linked to degradation, and in fact for CaMKII it has been shown to be a mechanism for activity modulation (26).

Second, HEK293T cells are not electrically active, meaning they do not generate the kinds of alterations in cytoplasmic calcium that activate neuronal CaMKII *in situ,* so activation of the WT kinase could not be tested directly in the intact cell. As a consequence, in this cell system, endogenous autophosphorylation is unlikely to be stoichiometrically significant since the half-lives of WT, T286/7A (unphosphorylatable at the autonomy site), and D135N (catalytically dead kinase, so cannot autophosphorylate) were not statistically different. These considerations further motivated the use of point mutants with locked activity-state confirmations.

Although N-terminal tagging of CaMKII for the most part has no consequences for activity or holoenzyme assembly (46), the use of fusion proteins also limits conclusions about absolute half-lives of the kinase variants. Importantly, the fact that all the kinase proteins we looked at had identical SNAP-tag fusions does allow us to make strong conclusions about relative half-lives and the influence of phosphorylation site mutations on degradation. Another consideration in the interpretation of half-lives is that the use of a cell line derived from human kidney tissue to look at the degradation of a fly protein makes conclusions about its absolute decay time tenuous. The lower relative ubiquitin labelling of dCaMKII compared to rCaMKII suggests that if degradation is mediated by the ubiquitin proteasome system, there may be some specificity of the mammalian system for activated mammalian CaMKII. The half-life of activated dCaMKII in a fly neuron with fly E3 ubiquitin ligases present may be closer to what we measure for rCaMKII in HEK293 (with the caveat for both that they lack neuronal context).

Nonetheless, the difference in half-life between the two proteins in HEK293T cells provided a useful way of determining what part of the rCaMKII protein was driving its activation-dependent degradation. The chimeric T287D protein that contained the rCaMKII catalytic domain was more unstable than dCaMKII, and the addition of the rCaMKII regulatory domain further accelerated degradation. This argues that the specificity is encoded in some feature(s) of both domains when they are in the active conformation.

What might be the basis of this specificity? Given that we and others have demonstrated that CaMKII can be ubiquitinated, there may be an E3 ubiquitin ligase that is capable of discriminating between the active and inactive forms of the mammalian kinase. Previous studies on the interaction of CaMKII with the ubiquitin proteasome system have demonstrated that ubiquitination can be a reversible activity modulator of the kinase in ischemic tissue (26). There have also been studies suggesting that CaMKII interacts with Ube3a both functionally (47, 48) and as part of a protein: protein interaction network (28). Whether this E3 is the one that is specific to the activated form of the kinase and signals its degradation is unknown, but given the clinical importance of dysregulation of CaMKII levels and activity (49) this will be important to determine. It is also possible that there will be multiple E3 enzymes capable of modifying CaMKII and having different functional outcomes (*e.g.* reversible inhibition *versus* proteolysis).

The failure to effectively ubiquitinate the activated dCaMKII in HEK293T may provide some important clues as to the molecular basis of activation-specific degradation. The lack of ubiquitination of dCaMKII might be due to a lack of binding of the relevant mammalian E3 to the fly enzyme catalytic/regulatory interface, or it may be due to differences in substrate lysines. Dhawka *et al.* (2024) identified a large number of modified lysines on CaMKII, and it is notable that three of the ones present in the mammalian catalytic domain (K32, K68, and K250) are not found in *Drosophila* CaMKII.

While our study is limited to cultured cells, the finding that it is the activated conformation of CaMKII that is most quickly degraded likely has functional implications for neurons. CaMKII is synthesized locally in neurons in response to activity in both *Drosophila* (1, 12) and mammals (11, 50). Unchecked activity-dependent accumulation could ultimately disrupt both plasticity and basal neuronal function by saturating potential interactors and/or disrupting synaptic structure by inducing inappropriate morphological changes in small synaptic spaces. Linking degradation to the active conformation of the kinase ensures that processes that induce new synthesis also stimulate CaMKII removal. How cells coordinate these processes to ensure that plastic changes are induced and maintained is not clear, but the control of both accumulation and degradation by activity ensures a level of homeostatic balance. Our identification of an activation state-dependent form of CaMKII degradation opens the door to a more nuanced understanding of these processes in the brain.

## Experimental procedures

### Cloning

The coding sequences of the PC isoform of *Drosophila* CaMKII (NP_524635) and *Rattus norvegicus* (NM_012920.1), a gift from the Lisman Lab at Brandeis University, were amplified *via* PCR from their respective plasmids and modified to include a 9-amino acid linker (GASGASGAS) at the N-terminal domain of the protein. The pSNAP-tag (m) Vector (#101135) obtained from Addgene was linearized using the PpUMI restriction enzyme. Overlap PCR was used to generate fragments, which were then inserted into the linearized vector using NEB Gibson Assembly Mastermix.

### Cell culture

HEK293T cells (obtained from ATCC) were cultured in complete DMEM, high glucose, with GlutaMAX supplement. Cells were seeded at a density of 2 × 10^5^ cells/ml 24 h prior to transfection. For transfection, 4 ug of plasmid DNA was added dropwise to 0.5 ml of 0.3 M CaCl_2_, pre-warmed to 37 °C. The DNA-CaCl_2_ mixture was then added dropwise to 2×HBS and mixed immediately. The resulting solution was added dropwise to a 10 cm plate of HEK293T cells. Media was replaced 6 h post-transfection to ensure optimal cell viability.

### Analysis and determination of half-life

SNAP::CaMKII constructs were expressed in HEK293T cells for 40 h. Cells were labeled with 1 μM TMR-Star (New England Biolabs, #S9105S) for 30 min at 37 °C, followed by three washes (15 min each) with 10 ml fresh media and a final 30-min wash. Equal volumes of labeled cells were split into a 6-well plate at a density of 1 × 10^5^ cells per well. Samples were collected at time points ranging from 4 to 96 h post-labeling, with at least five time points taken. Cells were resuspended in media, pelleted at 500×*g*, and washed twice with 1 ml PBS at 4 °C. The final pellet was stored at −80 °C for later processing.

Cell pellets were lysed in 100 μl mammalian cell lysis buffer (#GB-180–100) containing Halt Protease Inhibitor Cocktail (#78429) and Pierce Universal Nuclease for Cell Lysis (#88702). Lysis was performed using the Bioruptor Plus sonicator (10 min, 30 s ON/30 s OFF). Lysates were centrifuged at 14,000 RPM for 1 h at 4 °C. The supernatant was mixed with 4× Bolt LDS Sample Buffer and BME, then incubated at 70 °C for 15 min.

Processed lysates were analyzed using SDS-PAGE, and protein bands were visualized with the ChemiDoc Imaging System using the Alexa 546 filter. Protein bands were quantified using Bio-Rad Image Lab software 6.1, with normalized values fitted to a single-phase exponential decay curve (Y = A^xb^) using MATLAB 2017b. The fitted curves provided the decay rate, from which the half-life of the protein was calculated for comparison. Each biological replicate represents the half-life calculated from an independent transfection. Normality of the data for each comparison was assessed using the Shapiro-Wilk test.

Over the several years during which this study was conducted, we noticed that the absolute half-life of CaMKII was not completely consistent over different batches of cells and serum. Because of this, we include within the figure panels only data that were collected in the same set of experiments. This means that every control data set is an independent set of transfections with the WT kinase, and the comparisons between variants are made to a temporally matched control data set.

### Measurement of oligomerization state

Analytical gel filtration chromatography was performed using a Superose 6 10/300 Gl column (GE Healthcare) with a resolution range of 5 kDa to 5 MDa. The column was equilibrated in buffer containing 50 mM Tris (pH 7.5), 150 mM NaCl, and 1 mM TCEP at a flow rate of 0.4 ml/min.

A molecular weight (MW) calibration curve was generated using a mixture of protein standards: IgM (900 kDa), thyroglobulin (670 kDa), γ-globulin (158 kDa), ovalbumin (44 kDa), and myoglobin (17 kDa). The IgM standard was obtained from Rockland (chicken IgM; 003-0107), and the remaining standards were purchased from Bio-Rad (Gel-filtration standard; 1511901).

The partition coefficient (K_av_) was calculated for each standard protein using its retention volume, and K_av_ values were plotted against the Log_10_ of the MW from the Expasy MW calculator tool (51) to generate a standard curve. The resulting equation from this standard curve was used to estimate the MW of the SNAP::CaMKII constructs analyzed in this study. The calculated values are shown in Table 1.

SNAP::CaMKII constructs were expressed in HEK293T cells cultured in 10 cm plates. After 40 h, cells were labeled with 1 mM SNAP-Cell TMR-Star (TMR-Star) (New England Biolabs, S9105S), a cell-permeable fluorescent label, then harvested and lysed in a buffer containing 50 mM Tris (pH 7.5), 150 mM NaCl, 5 mM TCEP, Halt Protease Inhibitor Cocktail (#78429), and Pierce Universal Nuclease for Cell Lysis (#88702). Lysis was further facilitated using the Bioruptor Plus sonication device (10 min, 30 s ON/30 s OFF). The lysate was then centrifuged at 14,000 RPM for 1 h at 4 °C, and the supernatant was filtered using a 0.45 μm spin column to remove debris.

The cleared lysate was injected directly onto the gel filtration column without additional purification. The protein of interest was detected using the fluorescent signal emitted from TMR-Star, at an emission wavelength range of 580 to 590 nm. To normalize the data, a blank sample consisting of non-transfected HEK293T cells treated with TMR-Star under the same labeling conditions was used. For each construct, the area under the curve (AUC) for each sample was calculated using Prism software, allowing for comparisons between constructs.

### Calpain inhibitor experiments

SNAP::rCaMKII T286D was expressed in HEK293T cells for 40 h. Following the expression, cells were labeled with a mixture of 1 μM TMR-Star, and equal volumes of labeled cells were split into a 6-well plate at a density of 1 × 10^5^ cells per well. Cells were allowed to recover for 24 h before the initial samples were collected and stored at −80 °C. The experimental samples were treated with either DMSO or varying concentrations of Calpeptin (50 nM, 500 nM, and 5 μM). After an additional 24 h, the cells from each well were collected and lysed in 100 μl mammalian cell lysis buffer (#GB-180–100) containing Halt Protease Inhibitor Cocktail (#78429) and Pierce Universal Nuclease for Cell Lysis (#88702). Lysis was performed using the Bioruptor Plus sonicator (10 min, 30 s ON/30 s OFF). Lysates were centrifuged at 14,000 RPM for 1 h at 4 °C. The supernatant was mixed with 4× Bolt LDS Sample Buffer and BME, then incubated at 70 °C for 15 min. Processed lysates were analyzed using SDS-PAGE, and protein bands were visualized with the ChemiDoc Imaging System using the Alexa 546 filter. Protein bands were quantified using Bio-Rad Image Lab software 6.1 and normalized to the average initial signal. Each biological replicate represents the calculated fraction remaining from an independent transfection. Normality of the data for each comparison was assessed using the Shapiro-Wilk test.

### Detection of ubiquitination

SNAP::rCaMKII T286D was expressed in HEK293T cells for 40 h. Following the expression, cells were labeled with a mixture of 1 μM TMR-Star and 5 μM SNAP-Biotin (New England Biolabs, S9110S) labels. Cells were harvested and washed with PBS. Following this wash the cells were resuspended in PBS, and equal volumes were split into two Eppendorf tubes before pelleting. Both pellets are resuspended in 150 μl of lysis buffer containing 50 mM Tris (pH 7.5), 150 mM NaCl, 5 mM TCEP, 0.01% Triton X-100, Halt Protease Inhibitor Cocktail (#78429), and Pierce Universal Nuclease for Cell Lysis (#88702). For samples containing N-ethylmaleimide (NEM), a final concentration of 10 mM was used.

Lysis was performed using a Bioruptor Plus sonication device for 10 min, with cycles of 30 s ON and 30 s OFF. The lysates were clarified by centrifugation at 14,000 rpm for 1 h at 4 °C. The resulting supernatant was incubated with 125 μl of Pierce streptavidin agarose resin (Catalog #20353) for 30 min at room temperature with continuous rotation to allow binding of biotin-labeled proteins.

The unbound supernatant was removed by centrifugation at 500×*g* for 2 min, and the resin was washed six times with lysis buffer containing 0.1% Triton X-100 and excluding Nuclease. Each wash was performed by incubating the resin in the wash buffer for 10 min followed by centrifugation to remove the supernatant. Bound proteins were eluted from the resin by adding 1 × Bolt LDS sample buffer supplemented with 5% BME and heating at 70 °C for 15 min before centrifugation at 500*g* for 5 min.

Eluate from the streptavidin column was loaded onto an SDS-PAGE for separation and transferred to nitrocellulose membrane. The blot was probed using Anti-CaMKII-α 6G9 (1:1000, Cell Signaling #50049 and Anti-Ub (1:1000, Rockland #200-401-431), followed by secondary antibody application, Goat anti-Mouse IgG (H + L) Cross-Adsorbed Secondary Antibody, DyLight 680 (1:5000, Invitrogen #35519), Goat anti-Rabbit IgG (H + L) Cross-Adsorbed Secondary Antibody, DyLight 800 (1:5000, Invitrogen #SA5-10036), respectively. The protein bands were visualized with the ChemiDoc Imaging System using appropriate filters. Quantification of protein bands was performed using Bio-Rad Image Lab software 6.1.

Comparison of the T286D and T287D was done by separating the proteins in unpurified lysate from HEK293T cells using SDS-PAGE, followed by imaging of the TMR-Star signal with ChemiDoc Imaging System. Given the specificity of labeling, all bands with the TMR-Star signal are attributed to SNAP::CaMKll protein. Each normalized value of the high MW bands is from an independent transfection. Normality of the data for each comparison was assessed using the Shapiro-Wilk test. The MW of each band was estimated using the MW analysis tools within Bio-Rad Image Lab software 6.1.

### Statistical representation

For comparisons involving multiple data sets that were analyzed by ANOVA, we represent those differences using statistical groups (A, B, *etc.*). Each letter represents a group of data sets that were not significantly different (*p* > 0.05) from each other based on the *post hoc* test. Data sets labelled with different letters are significantly different (*p* < 0.05).

## Data availability

All data are contained within the manuscript.

## Conflict of interest

The authors declare that they have no conflicts of interest with the contents of this article.
